# LSTM Networks to Improve the Prediction of Harmful Algal Blooms in the West Coast of Sabah

**DOI:** 10.3390/ijerph18147650

**Published:** 2021-07-19

**Authors:** Fatin Nadiah Yussof, Normah Maan, Mohd Nadzri Md Reba

**Affiliations:** 1 Department of Mathematics, Faculty of Science and Technology, Universiti Teknologi Malaysia, Skudai 81310, Malaysia; fatinnadiah5@gmail.com (F.N.Y.); nadzri@utm.my (M.N.M.R.); 2 Faculty of Geoinformation and Real Estate, Universiti Teknologi Malaysia, Skudai 81310, Malaysia

**Keywords:** chlorophyll *a*, CNN, LSTM, prediction, satellite data

## Abstract

Harmful algal bloom (HAB) events have alarmed authorities of human health that have caused severe illness and fatalities, death of marine organisms, and massive fish killings. This work aimed to perform the long short-term memory (LSTM) method and convolution neural network (CNN) method to predict the HAB events in the West Coast of Sabah. The results showed that this method could be used to predict satellite time series data in which previous studies only used vector data. This paper also could identify and predict whether there is HAB occurrence in the region. A chlorophyll *a* concentration (Chl-a; mg/L) variable was used as an HAB indicator, where the data were obtained from MODIS and GEBCO bathymetry. The eight-day dataset interval was from January 2003 to December 2018. The results obtained showed that the LSTM model outperformed the CNN model in terms of accuracy using RMSE and the correlation coefficient *r* as the statistical criteria.

## 1. Introduction

Algal bloom is a phenomenon in which phytoplankton (algae) grows excessively in a river, lake, or sea. Harmful algal bloom (HAB) is a type of harmful algal bloom that causes toxicity in humans and marine life, resulting in negative impacts on the environment, human health, and economy. Environmental impacts include the death of marine organisms. Human health impacts include health deterioration as a result of the consumption of contaminated seafood, which can lead to serious illness or mortality. Economic impacts include the wreckage of tourism attraction spots, since activities such as fishing and snorkeling cannot be carried out, as well as the decline of the aquaculture sector due to enormous fish killings. For example, on 11 February 2014, due to the HAB event in Tanjung Kupang, Malaysia, the operators reported millions of ringgits of losses, affecting the fishery sector in Malaysia. Fish stocks, like snappers, cods, seabass, and threadfins, in nine farms were wiped out during the event [1]. In Penang, the aquaculture operators reported losses estimated at around RM20 million due to fish killings in 2005–2006 [1]. The growth in the frequency of HABs has alarmed the world over the decades, and it has become a major worldwide research topic among researchers [2,3,4,5,6,7,8].

Previous research has identified several parameters that influence the occurrence of HABs, but all of them require favorable environmental conditions, such as nutrient concentrations [9], light availability [10], water column stratification, or changes in water temperature [11]. Traditionally, the water sampling method was used in the affected areas for lab-based cell taxonomy. However, this conventional method is labor-intensive, and limits the scope of investigation in terms of space and time [12]. This contradicts the remote sensing-based detection method, which provides greater coverage, requires less time, and, most importantly, is less expensive. The chlorophyll *a* (Chl-a) concentration provides an indicator of eutrophication, as it measures the concentration of algae in a water body, and hence, it is used in remote sensing-based detection methods to detect HABs. The duration of HABs ranges from weeks to months, and they can cover up to thousands of square kilometers [13,14].

Only certain regions are covered by HAB forecasting systems, such as the National Oceanic and Atmospheric Administration (NOAA) HAB forecasting systems in the Florida region [15]. This system does not use machine learning (ML), and focuses solely on the human impact of HABs, such as respiratory problems and so on, with forecasts only lasting up to four days. HAB Observing System (HABSOS) is another system developed by NOAA [16] for HAB detection within the Gulf of Mexico.

The HAB events cause major concern due to their severe consequences to a variety of sectors. Prevention measures need to be taken for early detection for an appropriate HABs monitoring program to be constructed, and predictions need to be made to reduce the loss and damage caused by HABs [17]. From 2009 onwards, various predictive models have been successfully employed to predict HAB events, including the artificial neural network (ANN) [18,19,20], support vector machine (SVM) [12], random forest [13], neuro-fuzzy [21,22], and least square support vector machine (LSSVM) [23,24,25,26]. It is challenging to evaluate the HAB process, as it is a complex phenomenon with nonlinear factors. As a result, despite the outlined methods, a more advanced model is required to predict HAB events.

An advanced subfield of ML for artificial intelligence, known as deep learning (DL), has recently received considerable attention. Long short-term memory (LSTM) and convolutional neural network (CNN) are two popular models in DL [27]. LSTM is a recurrent neural network (RNN) that collects extended sequential data in the hidden memory for processing, representation, and storage. The constancy of time information is updated continually [28]. Meanwhile, the CNN’s structure consists of convolutional layers in which the neurons in the layers are connected to a small region of neurons in the input data. This is followed by sliding a weight matrix, called a filter, over the input and the convolution (or dot product) computed at each point, referred to as the feature map, between the input and the filter. This architecture enables the model to learn the filter needed to recognize identified patterns in the input data [29].

HAB data are represented by the concentration of chlorophyll *a* in the water body. In 2019, chlorophyll *a* concentration in water bodies was predicted using DL models. A CNN model was used to estimate chlorophyll *a* concentration in Daechung Lake, South Korea, with an emphasis on data skewness and imbalance [30]. The researchers indicated that using log transformation and oversampling techniques improved the performance of the CNN-based prediction model, particularly for small regions. Meanwhile, LSTM was applied for the multistep-ahead prediction of chlorophyll *a* in Gongju, South Korea [31]. The results revealed that the LSTM network model achieved higher accuracy than the dense network model and batch normalization, as a regularization method aided the learning process.

The prediction is significant because it can benefit the seafood and tourism industries, as well as the stakeholders involved. The major goals of this study are to (1) investigate the capabilities of DL models, namely the LSTM and CNN models, and (2) compare the performance of the LSTM and CNN methods in predicting HAB events. Previous research has explored both the LSTM and CNN methods, but no research has compared their performances in handling satellite data. Furthermore, to the best of the authors’ knowledge, this is the first time the CNN and LSTM models are used to predict HAB events on the West Coast of Sabah using satellite data.

## 2. Materials and Methods

### 2.1. Data

**Figure 1:**
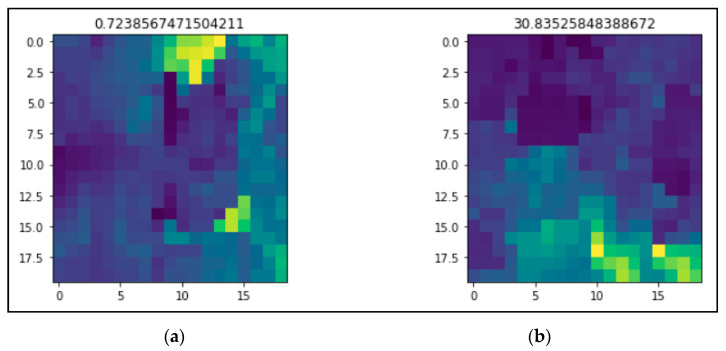
Plot of the modis pixel image that shows the maximum value highlighted on the top. (**a**) Maximum number in the modis pixel image is 0.7238567471504211 (**b**) Maximum number in the modis pixel image is 30.83525848388672.

**Figure 2:**
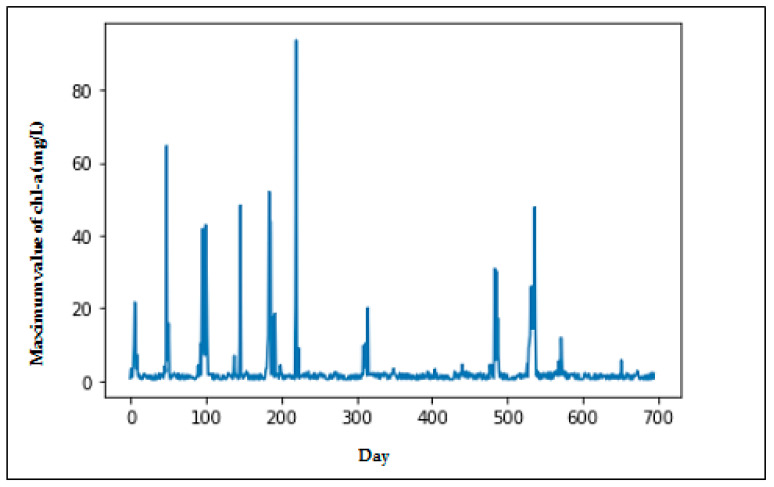
Plot of the maximum value of chlorophyll *a* concentrations.

Chlorophyll *a* from ocean color remote sensing was used to investigate phytoplankton dynamics, which marked the presence of HAB. MODIS-Aqua level 3 chlorophyll *a* data products of the reprocessed (2018.1) version were provided by the NASA Ocean Biology Processing Group (https://oceancolor.gsfc.nasa.gov) (accessed on 25 June 2021), as used in this study. The eight-day data period was extracted from January 2003 to December 2018 with a 1 km coverage and monthly resolution, and bounded by $4.6{\;}^{{}^{\circ}}N$ to $7.5{\;}^{{}^{\circ}}N$ latitude and $11.3{\;}^{{}^{\circ}}E$ to $16.5{\;}^{{}^{\circ}}E$ longitude. Due to cloud cover, there were spatial gaps in the data, which were filled using data interpolating empirical orthogonal functions (DINEOFs), an EOF-based technique. The image data captured by MODIS-Aqua were loaded into MATLAB and converted into matrix numbers, in which an image represents a matrix with a size of 20 × 19. The matrix numbers represent the covered region, and the maximum value in the matrix was selected to become a single datum. Our objective was to predict whether HABs occurred in the selected region. This could be accomplished by utilizing the maximum value in the matrix data for each image to identify HAB occurrence. The HAB threshold is 10 mg/L [32]. Figure 1 shows the modis pixel image for two subsequent datasets in February 2003 in which the maximum value of the pixel is highlighted on the top. Figure 2 illustrates the maximum value of chlorophyll-a concentration. It can be seen that there were several times that HABs occurred during the period from 2003 until 2018.

**Figure 3:**
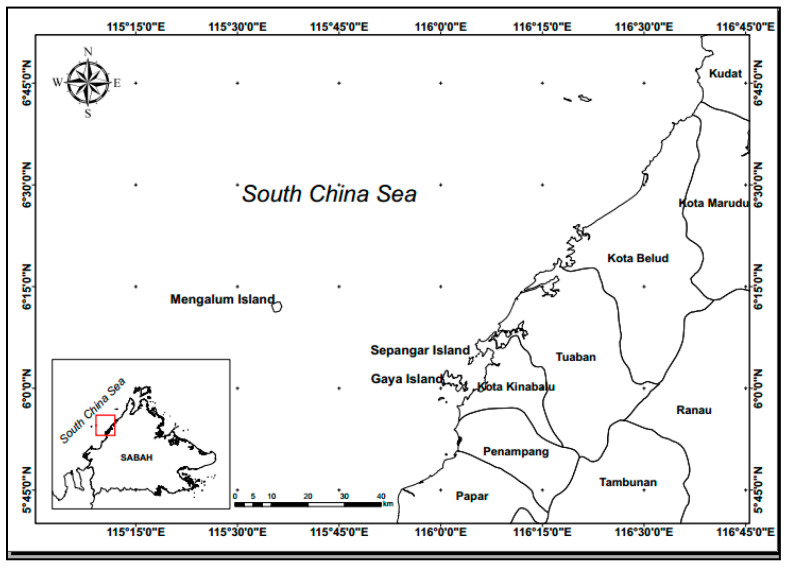
Location of the study area in the coastal waters of Kota Kinabalu, Sabah.

The study area was Sepanggar Bay, which is located on the coastal waters of Kota Kinabalu, Sabah (Figure 3). The freshwater inflows in this region come from the Inanam River and the Menggatal River, as well as factory waste and domestic sewage. The nearby inland is the terrestrial area, while on the southern side of Sepanggar Bay are reclaimed areas and a harbor. Gaya Island acts as a protective barrier on the bay’s northwestern side, while an aquaculture project for broodstock is located on the bay’s eastern side. Due to these reasons, the productivity of the phytoplankton varies temporally and spatially.

### 2.2. Long Short-Term Memory (LSTM)

**Figure 4:**
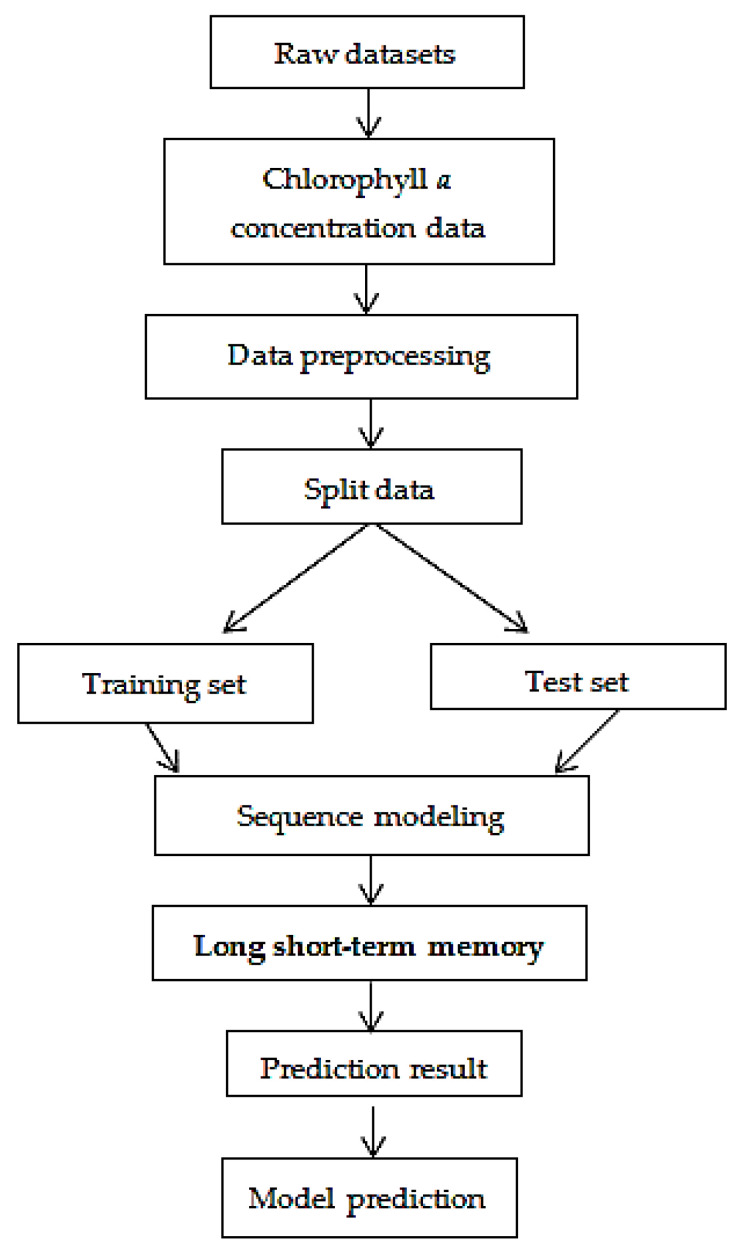
LSTM workflow for predicting harmful algal blooms (HABs).

The LSTM model, which is formulated from the recurrent neural networks (RNN) model, is capable of learning long-term dependencies, particularly changes in the hidden layer. There are three types of gates: the forget gate, input gate, and output gate. By examining the previous output and current input in the forget gate, the first sigmoid layer determines which information to discard from the cell state. A value ranging from 0 to 1 is set as a criterion for how much information to pass. A value of 0 indicates that no information is transmitted, whereas a value of 1 indicates that complete information is transmitted. The input gate determines whether the new information should be saved or discarded. Similar to the previous step, the sigmoid layer decides which value to update. Next, the tanh layer produces new results and combines the information from both layers to update the information. Finally, the output gate produces the output value by a filtered version.

(1) $$i_{t}=\sigma \left(W_{1}^{i}\times X_{t}+W_{h}^{i}\times H_{t-1}+b_{i}\right)\;\mathrm{Input}\;\mathrm{gate}$$

(2) $$f_{t}=\sigma \left(W_{1}^{f}\times X_{t}+W_{h}^{f}\times H_{t-1}+b_{f}\right)\;\mathrm{Forget}\;\mathrm{gate}$$

(3) $$o_{t}=\sigma \left(W_{1}^{o}\times X_{t}+W_{h}^{o}\times H_{t-1}+b_{o}\right)\;\mathrm{Output}\;\mathrm{gate}$$

(4) $${\tilde{C}}_{t}=\tanh\left(W_{1}^{C}\times X_{t}+W_{h}^{C}\times H_{t-1}+b_{c}\right)\;\mathrm{Cell}\;\mathrm{entrance}$$

(5) $$\mathrm{Sigmoid}=\frac{1}{1+e^{-1}}\;$$

(6) $$C_{t}=f_{t}\times C_{t-1}+i_{t}\times {\tilde{C}}_{t}$$

(7) $$H_{t}=o_{t}\times \tanh\left(C_{t}\right)\;$$

where $W_{1}^{i},W_{1}^{f},W_{1}^{o},W_{1}^{C}$ are the arrays of weights that connect $X_{t}$ to the three gates, $W_{h}^{i},W_{h}^{f},W_{h}^{o},W_{h}^{C}$ are the weight matrices that connect $H_{t-1}$ to all gates and cell entrance, $b_{i},b_{f},b_{o},b_{C}$ are the bias terms of all gates and the cell entrance, σ is the logistic sigmoid function, $C_{t}$ is the internal memory computed in this unit, *m* is a vector-only feature in every gate that obtains input from feature *m* of the cell vector, and $H_{t}$ is the output of a hidden state derived through memory multiplication. The LSTM architecture overcomes the vanishing gradient problem in the RNN. The detailed description of the LSTM algorithm used in our prediction is described in Table 1 and the LSTM workflow for predicting HAB is shown in Figure 4, where

C(t)= Internal memory of cell state
X(t)= Element wise input
H(t)= Output of the hidden state
H(t−1)= Previous hidden state
o(t)= Element of the output gate
i(t)= Element of input gate

### 2.3. Convolutional Neural Network (CNN)

The CNN is a method [33] that works well with images, particularly in image processing. Therefore, CNN is applicable for image forecasting time series. There are several layers in the CNN model, including the input layer, hidden layer, and output layer. A three-dimensional array input representing the height, weight, and number of channels is fed to a convolutional layer:

(8) $$a^{1}\left(i,h\right)=\left(W_{h}^{1}\times x\right)\left(i\right)=\sum_{j=-\infty }^{\infty }W_{h}^{1}\left(j\right)\times \left(i-j\right)\;$$

where

$$W_{h}^{1}\in R^{1xkx1}\;\mathrm{and}\;a^{1}\in R^{1xN-k+1xM_{1}}.$$

There is only one input channel, and the output of the first layer is then passed through the nonlinear activation function h(⋅) to produce $f^{\left(1\right)}=h\left(a^{1}\right)$. The hidden layer, unlike other neural networks, is made up of a convolutional layer, a pooling layer, and a fully connected layer. The convolutional layer is related to the feature extraction of raw input with learnable filters [34]. Filters, also known as kernels, depict a specific feature. The CNN operates the convolution using a weight-sharing concept, which reduces the number of parameters. The weights are constantly updated during the training process [35]. The pooling layer reduces the feature dimension by producing one value out of all of the pooling window values. This reduces the input layer with a max-pooling operation, where the maximum value of the previous layer is selected [34], and aids in overcoming the problem of computational cost and overfitting [35].

The input feature map $f^{l-1}\in R^{1xN_{l-1}xM_{l-1}}$ located in the hidden layer l=2,…,L is convolved with a set of filters $W_{h}^{1}\in R^{1xkxl},h=1,\ldots ,M_{1}$ denoted as $M_{1}$ to produce a feature map $a^{1}\in R^{1\times N_{1}\times M_{1}}$ as below [29]:

(9) $$a^{1}\left(i,h\right)=\left(W_{h}^{l}\times f^{l-1}\right)\left(i\right)=\sum_{j=-\infty }^{\infty }\sum_{m=1}^{M_{l-1}}W_{h}^{1}\left(j,m\right)f^{l-1}\left(i-j,m\right)\;$$

**Figure 5:**
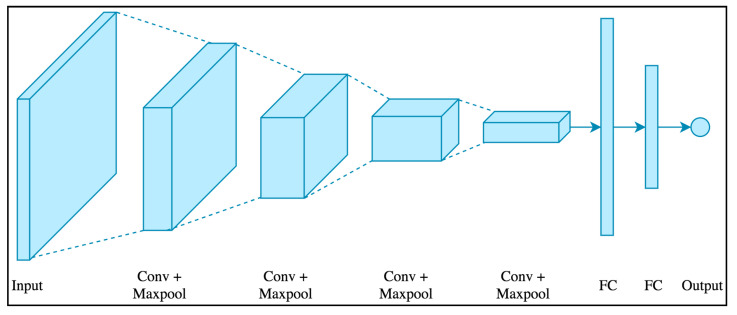
The CNN model architecture.

The high dimensions of the feature extracted are flattened by the fully connected layer and incorporated to get the final output:

(10) $$l_{t}=\tanh\left(X_{t}\times k_{t}+b_{t}\right)\;$$

where $l_{t}$ is the output value after convolution, *tanh* is the activation function, $X_{t}$ is the convolution kernel’s weight, and $b_{t}$ is the bias of the convolution kernel. The architecture of the CNN model is shown in Figure 5.

### 2.4. Evaluation Criteria

The accuracy of the methods is evaluated using the Pearson correlation coefficient (*r*) and root means square error (RMSE). In Equation (11), *x* represents the observed value, *y* represents the forecasted value, and $\bar{x}$ and $\bar{y}$ are the means of *x* and *y,* respectively. The formula of the Pearson correlation coefficient is as follows:

(11) $$r=\frac{\sum \left(x-\bar{x}\right)\left(y-\bar{y}\right)}{\sqrt{\sum {\left(x-\bar{x}\right)}^{2}{\left(y-\bar{y}\right)}^{2}}}\;$$

In Equation (12), there are *n* missing data points in the test datasets, where $Y_{act}^{i}$ is the actual value for the ith missing data point and $Y_{est}^{i}$ is the missing data point estimated value.

(12) $$RMSE=\sqrt{\frac{\sum_{i}^{n}\left(Y_{act}^{i}-Y_{est}^{i}\right)}{n}}$$

### 2.5. Training LSTM Network

One LSTM network was trained independently at a time using each one-dimensional time series corresponding to a pixel of the stacked images in the time axis. The whole satellite image time series with 640 time steps from 1 January 2003 to 31 December 2018 was divided into two partitions for data preparation: 70% for the training stage and 30% for the testing stage. The training stage was used to develop the models, while the testing stage was used to validate and compare the performance of the models in the training phase. Chl-a concentration (mg/L) was used as the sole model input data, since using a single variable as the input provides a higher accuracy than using many variables as inputs [36]. A normalization procedure via minimum–maximum scaling techniques was applied to the input and output variables scaled between 0 and 1 to prevent sudden gradient changes and smooth the convergence.

Several samples were obtained using the sliding time window-based method [37] to train the LSTM network from a single historical datum. For example, suppose the time window size is *l > n* or a time series $\left(x_{1},x_{2},\ldots ,x_{n}\right)$ with *n* time steps, then the first input subsequence for output $x_{l+1}$ is $\left(x_{1},x_{2},\ldots ,x_{l}\right)$. A training sample is generated by sliding the time window one time step ahead at a time. The one time step ahead output $x_{t}$ corresponds to the subsequence $\left(x_{t=1,\ldots ,}x_{t-2},x_{t-1}\right)$ in the time window. Therefore, training the LSTM network could produce samples *n* − *l* + 1.

### 2.6. Prediction Based on Trained LSTM

The LSTM can only predict one time step at a time. Thus, the output $y_{t+1}$ uses an input sequence $\left(x_{t-l+1},\ldots ,x_{t-1},x_{t}\right)$ with an *l* step sliding time window, while the output $y_{t+n}$ is predicted using the input sequence $\left(x_{t+n-l,\ldots ,}x_{t},y_{t+1,\ldots ,}y_{t+n-1}\right)$. This corresponds to a strategy to implement the prediction of *n* steps iteratively using the predictive outputs of the previous steps to compose the input sequence to the next step. Hence, the input sequence $\left(x_{t+n-l},\ldots ,x_{t},y_{t+1},\ldots ,y_{t+n-1}\right)$ is used to predict the output $y_{t+n}$.

The input layer consisting of Chl-a concentration was fed to all three layers, including their lag times. The rectified linear unit (ReLU) is a piecewise linear function that returns output directly when positive and zero otherwise. Unit number 64 was used in the hidden layer, and a dropout layer with a rate of 0.001 was used after the LSTM layer to prevent overfitting and underfitting. Overfitting occurs when a network is overtrained and loses generality on the test dataset, whereas underfitting occurs when a network is poorly trained and the pattern remains unrecognized. The linear activation function and the fully connected layer, called “dense”, were applied with a unit of 1. Then, the model was compiled with an RMSE loss function and an adaptive gradient algorithm (AdaGrad) optimizer with a learning rate of 0.01. All of the components used were supported by functions of the Keras library in Python.

### 2.7. Prediction Based on the CNN

The CNN model was trained with a hidden layer, where a convolution layer (Conv1D) with a filter size of 64, a kernel size of 1, the same padding type, a ReLU activation type, and a uniform kernel initializer were used. Furthermore, a pooling layer with max pooling and a pooling size of 1 was applied with the maximum value from each neuron cluster in the previous layer. To overcome overfitting, a dropout layer with a rate of 0.001 was used after the CNN pooling layer [38]. A fully connected layer, called “dense”, was applied to the output layer, which used a linear activation function. Finally, the model was compiled with an RMSE loss function and an AdaGrad optimizer with a learning rate of 0.01. Similar to the LSTM method, all of the components used were supported by functions of the Keras library in Python.

## 3. Results

The results are summarized in Table 2. In the training and testing phase, the LSTM model, which had an r = 0.338386 and RMSE = 3.402142 mg/L, outperformed the CNN model, which had an r = 0.111790 and RMSE = 4.361724 mg/L. These findings are backed by previous research [38,39] that claimed that the LSTM model outperformed the CNN model.

**Figure 6:**
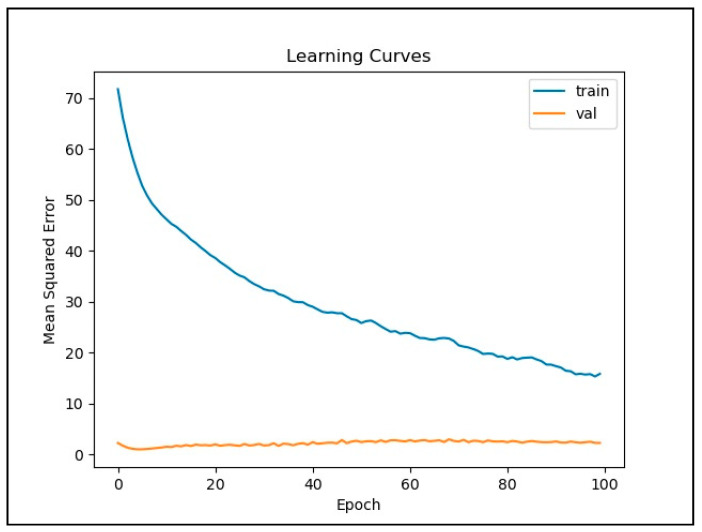
Loss function plots for the LSTM model.

**Figure 7:**
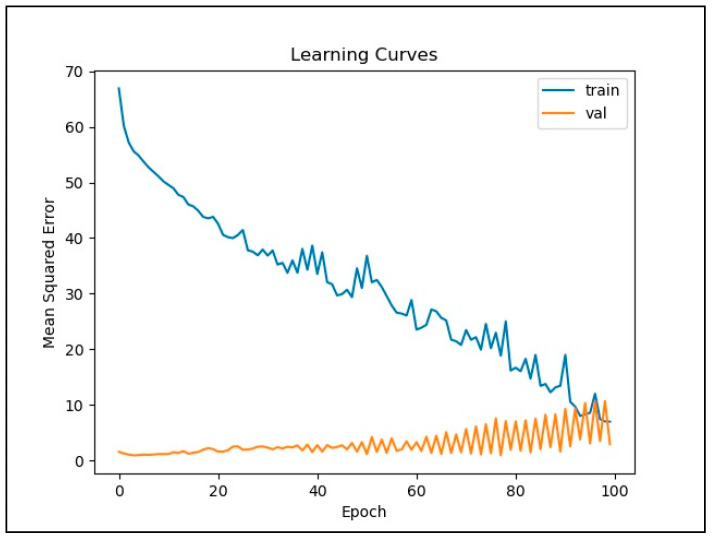
Loss function plots for the CNN model.

Figure 6 and Figure 7 illustrate the loss function plots of the LSTM and CNN models, respectively. In Figure 6, the graph of the loss function plots configuration illustrates that overfitting and underfitting were avoided in the LSTM model, since the curve of validation loss was always below the curve of training loss. The curve of the training loss function in the LSTM graph shows a steady improvement in the prediction quality throughout the training process. Meanwhile, the validation loss curve shows no further improvement after a small number of epochs. In contrast, the CNN loss function graph in Figure 7 shows that overfitting occurred when the number of iterations reached its maximum. This exemplifies that the CNN was incapable of capturing data with a long-term dependency.

**Figure 8:**
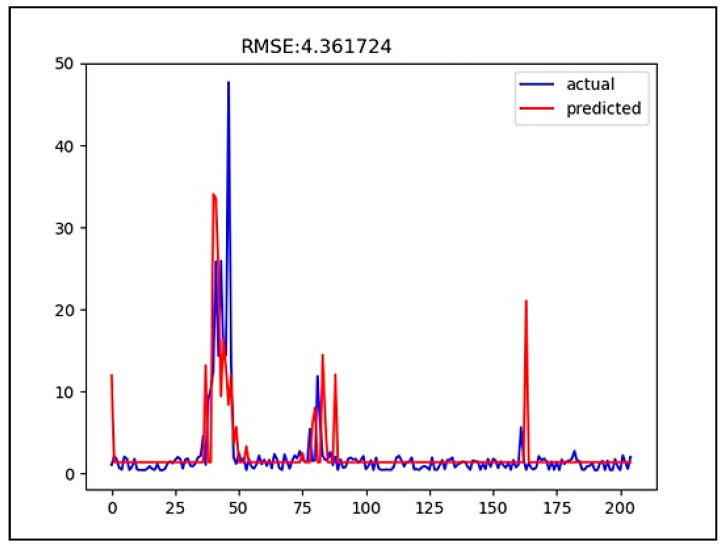
Comparison of predicted and actual values in predicting HABs using the CNN method.

**Figure 9:**
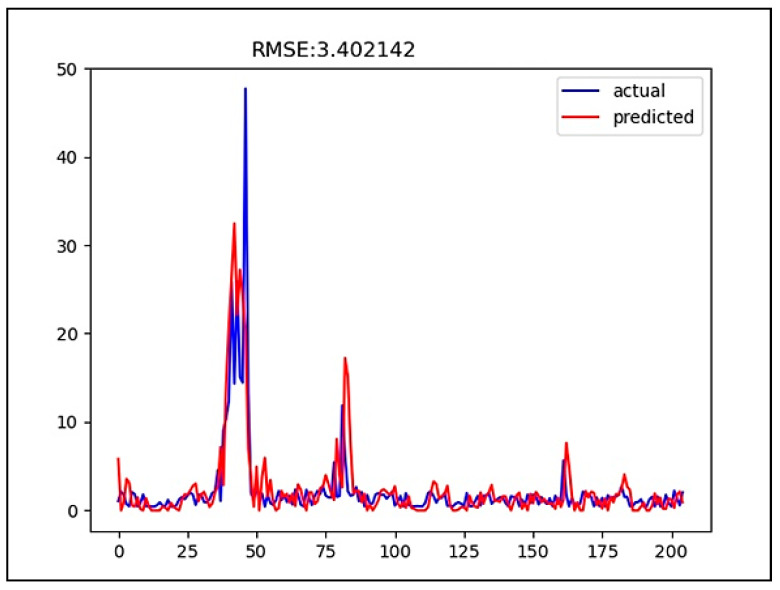
Comparison of predicted and actual values in predicting HABs using the LSTM method.

Figure 8 and Figure 9 show the graphs of predicted and actual values in predicting HABs, respectively. The blue line is the actual data, while the red line is the predicted data. In this study, chlorophyll-a data were predicted eight days in advance. During the training, it could be seen that an HAB occurred within the period in which the value of the concentration of chlorophyll a was greater than 10 mg/L.

**Figure 10:**
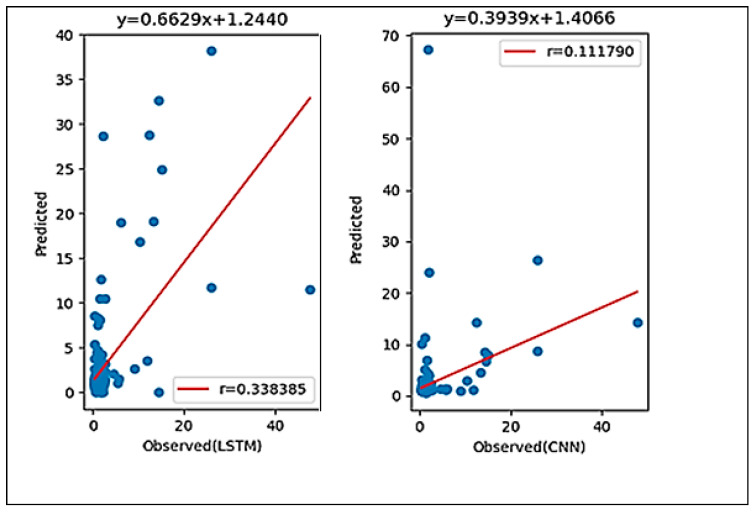
Plots of the observed and predicted Chlrophyll *a* concentration using the LSTM and CNN models.

Figure 10 shows the scatter plots of both models, demonstrating that the LSTM model had a higher *r* and was well-described by linear regression (y = 0.6229x + 1.2440). This indicated that the LSTM model was more accurate than the CNN model, with y = 0.3939x + 1.4066. Both methods had a positive correlation coefficient *r*, indicating that, as x increased by one point, y increased by the coefficient value.

## 4. Discussion

From the results, the LSTM method outperformed the CNN method in terms of the accuracy of r and the RMSE. These findings are backed by previous research [38,39] that claimed that the LSTM model outperforms the CNN model. This is due to the CNN model’s inability to capture long-term dependencies, while the LSTM model can effectively capture long temporal dependencies. LSTM model algorithms were designed to solve the problem of information loss for long-term memory in existing RNN models, which is known to improve predictions by transferring information on previous data as the amount of data grows [23].

However, the r value is insufficient in demonstrating that the LSTM method is the best at predicting HAB events. This is due to the missing data problem, since there is the limitation of using satellite data where there is always clouds cover. The result is improved, since the missing data problems were solved using the DINEOF method [40] instead of omitting it [23]. Therefore, the purpose of this study is not to propose a new forecasting methodology, but rather to aid in the discovery of relevant approaches for predicting HAB events.

The significance of this study is to show that the LSTM model could be employed to predict HABs, since it has the ability to handle long-term dependency data. Since HABs occur annually in the West Coast of Sabah, this prediction helps to give early signals to the authorities and the region community on the occurrence of HABs. Therefore, several preventive measures can be done to cater to the adverse effects of HABs. For example, associated authorities, like the Ministry of Health, could give early warnings to the public against eating shellfish during that period to prevent the ingestion of toxic seafood that could affect human health.

It was discovered that DL models have greater flexibility in learning nonlinearity, particularly in handling large datasets. Many previous studies have demonstrated the capabilities of the CNN and LSTM models in solving short-term predictions [23,39]. In this study, the performance of the LSTM method demonstrates the capability to improve the short-term prediction accuracy model for algal blooms. The predictions eight days in advance would help to implement several preventive measures for algal bloom mitigation.

## 5. Conclusions

The LSTM and CNN models were used to predict HAB events on the West Coast of Sabah based on satellite data and chlorophyll *a* concentration. The data were collected from 1 January 2003 to 31 December 2018, and were divided into two partitions: 70% for training and 30% for testing. Statistical criteria, such as the RMSE and correlation coefficient r, were used to evaluate the testing period performance. The results showed that the LSTM model outperformed the CNN model across all statistical criteria, since the LSTM method can learn long-term dependencies, which the CNN method cannot. However, the correlation coefficient r of the LSTM model is still low and not very strong. For future work, studies on improving the value of r using other appropriate forecasting methods shall be conducted, such as using a hybrid model that combines two methods to improve the efficiency of the model. In addition, it is also suggested to study how to input the matrix data into the prediction models without having to transform into vector data.

There are some drawbacks using the LSTM model, where different random weight initialization affects this method. Instead, the model prefers small weight initialization. Not only that, but the LSTM is also prone to overfitting, and the dropout algorithm is difficult to apply to curb this issue. In addition, there are also some limitations in using satellite time series data in which there are always missing data, since there is cloud cover. Therefore, it becomes a limiting factor to obtain an accurate prediction of HABs even when the data have been corrected. Satellite data also could not identify the species blooming in that time. In other words, HABs could be predicted without knowing the species involved. In this regard, it is recommended to explore the capability of other DL models for medium- and long-term prediction in future research, since longer-term prediction is more challenging, because it requires more pertinent historical input data than short-term prediction [32].

## Figures and Tables

**Table 1:** LSTM procedures.

Step	Description
1	Preprocessing of all fine particulate matter and meteorological data LSTM pre-training
2	Denote $X_{\left(t\right)}$ as an element-wise input, ignoring bias *W* is created, which represent the weight matrix to transfer the input from cell to gate vectors Elements in the gate vector are defined as *m* and receive information from the cell state The internal memory of the cell state is denoted as *C*
3	Fine-tuning Build a vector by applying the tanh function to the cell state Apply the sigmoid function to create a filter for values of $H_{t-1}$.
4	Obtain prediction results

**Table 2:** Comparison of the prediction accuracy of the long short-term memory (LSTM) model with the convolution neural network (CNN) model.

Model	RMSE	*r*
LSTM	3.402142	0.338385
CNN	4.361724	0.111790

## Data Availability

Data available in a publicly accessible repository that does not issue DOIs. Publicly available datasets were analyzed in this study. This data can be found here: [https://oceancolor.gsfc.nasa.gov] accessed on 15 July 2021.
